# ActiviTeen: A Protocol for Deployment of a Consumer Wearable Device in an Academic Setting

**DOI:** 10.2196/resprot.5934

**Published:** 2016-07-25

**Authors:** Alexa M Ortiz, Stephen J Tueller, Sarah L Cook, Robert D Furberg

**Affiliations:** ^1^ RTI International Research Triangle Park, NC United States

**Keywords:** mHealth, clinical research protocol, Fitbit, physical activity tracker, survival analaysis, technology deployment, education

## Abstract

**Background:**

Regular physical activity (PA) can be an important indicator of health across an individual’s life span. Consumer wearables, such as Fitbit or Jawbone, are becoming increasingly popular to track PA. With the increased adoption of activity trackers comes the increased generation of valuable individual-based data. Generated data has the potential to provide detailed insights into the user’s behavior and lifestyle.

**Objective:**

The primary objective of the described study is to evaluate the feasibility of individual data collection from the selected consumer wearable device (the Fitbit Zip). The rate of user attrition and barriers preventing the use of consumer wearable devices will also be evaluated as secondary objectives.

**Methods:**

The pilot study will occur in two stages and employs a long-term review and analysis with a convenience sample of 30 students attending Research Triangle High School. For the first stage, students will initially be asked to wear the Fitbit Zip over the course of 4 weeks. During which time, their activity data and step count will be collected. Students will also be asked to complete a self-administered survey at the beginning and conclusion of the first stage. The second stage will continue to collect students’ activity data and step count over an additional 3-month period.

**Results:**

We are anticipating results for this study by the end of 2016.

**Conclusion:**

This study will provide insight into the data collection procedures surrounding consumer wearable devices and could serve as the future foundation for other studies deploying consumer wearable devices in educational settings.

## Introduction

Regular physical activity (PA) is an important predictor of physical health across the life span [1]. Nonetheless, the frequency of inactivity continues to be problematic for a large number of children and adolescents [2-4]. Although methods for objectively measuring PA in children and adults in naturalistic settings are well established (ie, accelerometry), they are nearly exclusively used in research contexts due to cost and technical requirements that impede their wide-scale use. There does not yet exist low cost, low burden, scalable approaches for the objective measurement of PA. Ultimately, this undermines surveillance efforts by governmental or regulatory agencies, efforts to evaluate PA-related changes in public policy, and schools’ abilities to evaluate the effectiveness of their PA-related interventions.

Wearable devices available to consumers offer a wide range of features and can be used to monitor an individual’s sleep, diet, or PA. A 2014 national survey indicated that out of 1000 US consumers, one in five individuals own a wearable and one in ten individuals use their wearable on a daily basis [5]. Additionally, given that 64% of Americans own a mobile phone with app capabilities (smartphone) [6], more and more users now own the necessary technology to connect their mobile phone directly to their wearable. Consequently, the increased adoption of consumer wearable devices has led to a concomitant rise in the quantity of individual-based generated data. Collection of this data could potentially provide valuable insight into an individual’s daily routines, lifestyle, and behaviors.

Despite the boom in adoption of consumer wearables, the devices also appear to have a high rate of user abandonment. Research has shown that more than half of activity tracker owners no longer use their device and a third of owners stop using their activity tracker within six months after receiving the device [7]. Moreover, there might be a wide range of factors preventing long-term use of consumer wearables. Not providing new information to the user, inaccurate tracking results, discomfort, or an unpleasing aesthetic design are all barriers that may prevent continued use of consumer wearable devices [7-10].

To assess the practicality of data collection employing consumer wearables with a sample of high school students, the ActiviTeen study was developed. ActiviTeen is a pilot study with a long-term approach to data collection employing a single type consumer wearable device (the Fitbit Zip). This tracking approach offers the benefit of passively collecting data using devices that people typically purchase for their own use to achieve quantitative and unobtrusive data collection for an important component of their health outcomes: PA. The primary aim of ActiviTeen is to demonstrate the feasibility of data collection from the designated consumer wearable devices. Secondary outcomes consist of assessing the rate of attrition study participants experience with wearable devices and identifying barriers preventing participants from routinely using their wearable devices. For the purpose of this paper, the term “Fitbit Zip” and “Fitbit” will be used interchangeably.

## Methods

### Device Description

The Fitbit Zip was selected due its good reliability and validity and it has shown a significant correlation as an indicator of PA when compared to the Actigraph accelerometer and the Yamax mechanical pedometer [11,12]. The Fitbit Zip will provide data related to the user’s step count and level of activity. The participant’s step count will consist of the number of steps taken per day. Activity data is calculated by utilizing metabolic equivalents (METs) [13]. The Fitbit will estimate the user’s MET value based on the intensity of their activity [13], this will then be broken down into daily minutes sedentary, daily minutes lightly active, daily minutes fairly active, and daily minutes very active.

### Participant Eligibility Criteria

ActiviTeen will use a convenience sample of students recruited from Research Triangle High School, located in Durham, North Carolina. Each of the following criteria must be met in order for students to be considered eligible for participation: students must be between 13 and 17 years of age, able to speak and read English, and students must be agreeable to having their step count and activity level monitored throughout the entirety of their study participation. In addition, eligible students must maintain ownership of an Apple or Android mobile phone with app capabilities (smartphone) throughout the study. The mobile phones (smartphones) should be capable of connecting to a wireless network and downloading the free Fitbit mobile app from either iTunes or Google Play. Mobile phone ownership among teenagers is 78%, and of those, 47% own a mobile phone with app capabilities (smartphone) [14]. Therefore, mandating mobile phone (smartphone) ownership as an eligibility requirement is not anticipated to be problematic.

### Recruitment Procedure

ActiviTeen’s population will consist of up to 30 student participants. The recruitment of 30 students is feasibly managed by the research team, but still allows for sufficient data to be collected regarding Fitbit usage. For recruitment, the research team will run a brief description of the study within the school’s student/parent newsletter to identify participants. The description of ActiviTeen will review the following key points: (1) the purpose of ActiviTeen, (2) student eligibility criteria,(3) emphasis that participation in ActiviTeen is voluntary, (4) emphasis that participation will not impact students’ academic standing or be a source of extra credit, and (5) that study recruitment is on a first-come, first-serve basis. The newsletter will also state that students will be permitted to maintain ownership over their assigned Fitbit Zip once the study concludes, provide the email contact for a member of the research team and the date when ActiviTeen recruitment will end. Students will be instructed through the student/parent newsletter to email a member of research team if interested in study participation.

### Trial Design

**Figure 1:**
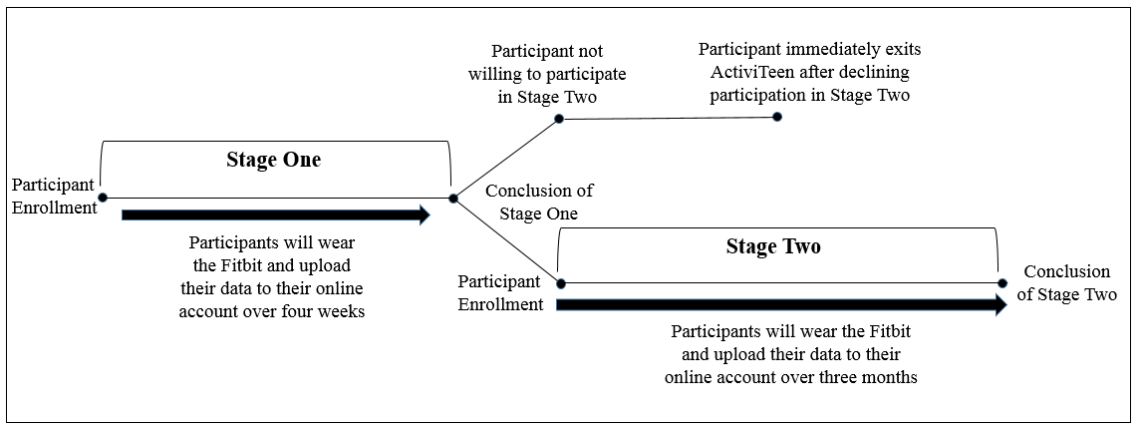
ActiviTeen design.

ActiviTeen is an exploratory, pilot study occurring over two stages, as outlined in Figure 1. Since a third of US consumers who own an activity tracker stop using their device in six months [7], the original study design took place over a 6-month period. However, the length of the study later had to be tailored to 4 months to align with the school’s academic calendar, allowing the study to conclude before students were dismissed for summer break. It was also hypothesized that the most significant decline in use would be observed in the first month, leading to the creation of a 1-month check-in. Although a formal exit point is outlined upon the conclusion of Stage 1, participation is voluntary and students are permitted to leave the study at any point during the two stages outlined below.

### Intervention

#### Enrollment Preparation

Prior to distribution of the Fitbits, the research team will assign each study participant a unique ID. Each ID will be associated with a separate online account created through the Fitbit website. To simplify the device set-up process, the 30 individual Fitbit accounts will all be associated with one email address: “ActiviTeenRTI@gmail.com”. This will be completed by adding a “+” and the student’s participant ID to the original email address above, creating 30 individual addresses with the following format: “ActiviTeenRTI+101@gmail.com”. While the Fitbit site will view all email addresses separately, Google will not recognize the “+” or the numbers following the “+” as part of the email. Consequently, this allows for the 30 Fitbit accounts to simultaneously link back to a single email address.

Thirty individual packets will then be compiled containing the student assent, an ActiviTeen information sheet, an ActiviTeen frequently asked questions document, the Fitbit Zip product manual, the pre-data collection survey, and a check-off list for the research team member to review when enrolling the student. Each participant ID will be associated with one packet, one online Fitbit account, and one Fitbit Zip.

The first 30 students who respond to the newsletter and meet all eligibility requirements will receive a recruitment email stating when the research team will arrive at Research Triangle High School to enroll students and distribute the Fitbits. Those students who respond to the newsletter, but do not meet eligibility requirements, will be sent an email thanking them for their interest and stating they are unable to participate. Students who contact the research team once 30 participants have been successfully recruited will be sent an email thanking them for their interest and stating no further students can be recruited since ActiviTeen has reached the max capacity of participants.

#### Participant Enrollment

**Figure 2:**
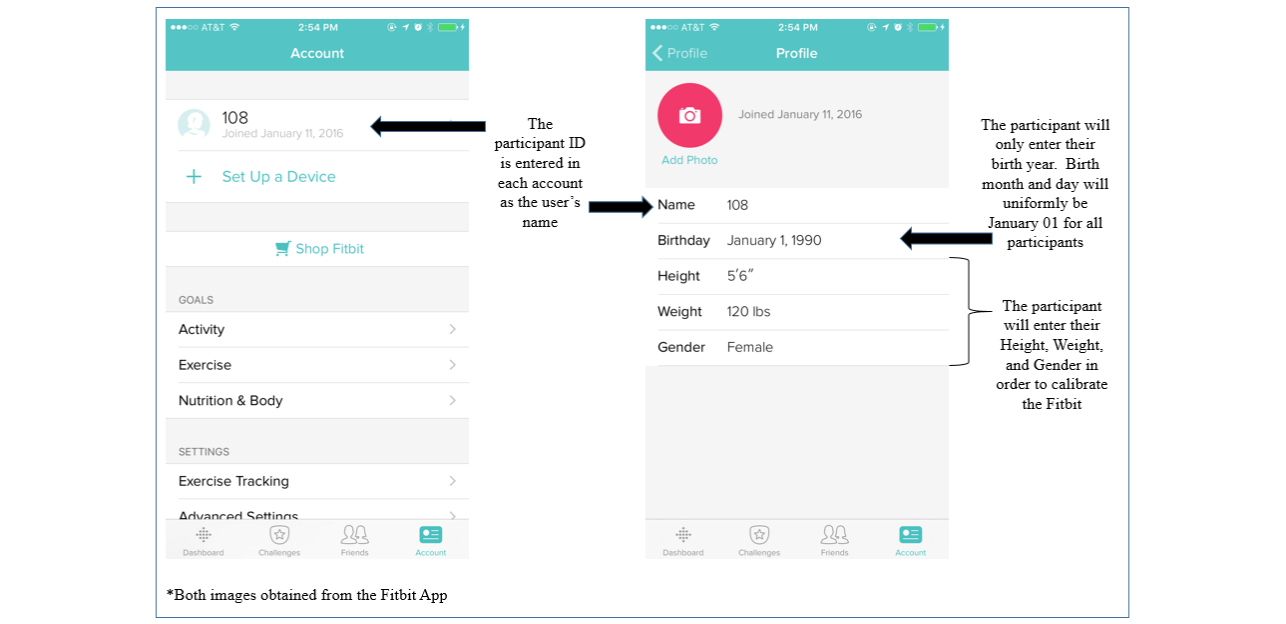
Fitbit mobile app appearance.

To begin participant enrollment, a member of the research team will individually review each document within the compiled packet and have the participant sign the student assent. Afterward, the student will download the Fitbit app onto their mobile phone and log into one of the previously created Fitbit accounts. To initially calibrate the Fitbit Zip each student will enter their gender, height, and weight. Birth year will be collected by Fitbit, but participants will be instructed not to enter the month or day they were born. To ensure anonymity, January 1st will be uniformly entered as the birth month and day for all participants. The view of how the app will appear on an iPhone is provided in Figure 2. While there are some formatting differences between iPhones and Androids within the Fitbit app, the majority of data elements in Figure 2 are similar across the two device types. Additional features, such as logging calories consumed and creating a food plan, are offered by the Fitbit app and will not be disabled for the study. Students will be instructed not to record any additional information beyond the activity data and step count automatically recorded by their Fitbit device. However, if students do choose to utilize the supplementary features during the study, the additional data will not be reviewed or downloaded by project staff. If unnecessary data beyond the students’ activity data and step count is downloaded, it will be immediately deleted. Upon receiving the Fitbit Zip devices, students will also be instructed they are not financially responsible for the device if lost, stolen, or damaged throughout the study. A second device will not be provided if the first becomes lost or unusable due to actions on the part of the student; however, the research team will replace Fitbit batteries and faulty devices as needed.

Students that are absent during enrollment for ActiviTeen will be permitted to enroll at a later date upon notification of the project staff. Although the student might enroll a few days after their peers, they will continue to follow the pre-designated study timeline and will not be given additional days of data collection. However, it will be the student’s responsibility to notify project staff via email of their absence and continued desire to participate in the study. Students who do not show up during the enrollment period for stage one or stage two of ActiviTeen will not be re-contacted regarding participation.

### ActiviTeen Stage One

At the beginning and end of ActiviTeen’s first stage, student participants will also be asked to participate in a 10-minute self-administered paper-based survey. The pre-data collection survey will be provided to students as part of their enrollment and will ask the participant if they plan to change their behavior knowing they are being monitored, is it likely they will forget to wear their Fitbit, and do they foresee any problems attaching the Fitbit to their clothes? The post-data collection survey will be administered after the first stage of ActiviTeen has completed and will ask the participant if they were able to wear the Fitbit for the entire 4-week period, how often they forgot to wear their Fitbit, was the Fitbit lost or damaged, were there any problems attaching the Fitbit to their clothes, and if they would be interested in participating stage two of ActiviTeen? Both the pre- and post-data collection surveys will ask the participant their grade level and gender.

For the first stage of ActiviTeen, each Fitbit Zip will be worn by one student participant on their right hip over 4 weeks. The hip was chosen for placement due to the device’s enhanced accuracy for counting steps [15]. Students will be instructed during enrollment to wirelessly sync their Fitbit Zip with the downloaded Fitbit app every 3-4 days, transmitting their activity data to their online Fitbit account. To avoid excessive cellular data usage, students will be given the option during recruitment to disable the “All-Day Sync” function offered in the Fitbit app. It will not be mandatory for students to use or disable this feature. In addition, while the students’ Fitbit will sync with their online account when the Fitbit app is open and within range of the device, the students will not be reminded by project staff to sync their device at any point throughout the study. The research team will monitor each student's account over the 4-week data collection period and collect the student's activity data and step count by downloading the recorded activity data directly from the online Fitbit portal on a weekly basis.

### ActiviTeen Stage Two

Once the initial 4 weeks has concluded, each student will receive an email stating when the ActiviTeen research team will return to campus. During the second visit to Research Triangle High School, student participants will be asked to complete the second paper-based post-data collection survey. Students will then be given the option to continue wearing the Fitbit Zip and allow the research team continued access to their activity data for an additional 3-month period (the second stage of ActiviTeen) or decide to decline continued participation. If they decide not to continue in the study, the student will exit the study and their Fitbit will be removed from the research team’s previously created account. For those students who elect to remain in the study, they will be asked to sign a second student assent document and be reminded to continue wearing the device on their right hip and continue syncing their activity data every 3-4 days. The research team will continue downloading the participant’s activity data and step count on a weekly basis. When the second stage of ActiviTeen concludes, the remaining student participants will receive an email stating the study has concluded and their Fitbit Zip has been disconnected from any previously made account. ActiviTeen will not utilize strategies to improve participant adherence. However, upon exit of the study, all students will be given their assigned Fitbit Zip and will be allowed to create and personalize their own Fitbit account. No survey will be given to student participants upon the conclusion of the second stage. Deploying a survey in the second stage would likely yield very few respondents given the potential loss to participant attrition. Consequently, it was decided the focus should be active data collection in the first stage to optimize response rates.

### Informed Assent

Due the ActiviTeen’s minimal risk, a waiver of signed parental consent was received from the RTI International Institutional Review Board (IRB). However, an ActiviTeen information sheet providing a general overview of the study will be emailed to the parents/guardians of all student participants. Before distributing the Fitbit Zip for the first stage of data collection, students will be asked to review and sign an assent form. Once the student’s assent is signed, a verbal confirmation will be obtained to conduct a brief pre-data collection survey. After the initial 4 weeks of data collection has concluded, the research team staff will obtain verbal confirmation from the student to conduct a brief post-data collection survey. If the student is willing to participate in the second stage of data collection, a second assent form will be reviewed and signed.

The assent documentation for both stages of the ActiviTeen study provides an overview of the study as well as how data collection will occur. In addition, the assent addresses the student survey, reminds the student that their participation is voluntary, and reviews benefits as well as risks of participation. The documents also explain how the student’s privacy will be protected and who they or their parent/guardian can contact for any follow-up questions or concerns.

### Outcome Measures

ActiviTeen’s primary outcome is to establish the feasibility of data acquisition from the designated consumer wearable device, the Fitbit Zip. Ease of downloading and collecting participant’s activity data and step count throughout the study will be a vital determinant. The secondary outcomes will be an assessment of the rate of attrition study participants experience throughout the course of the study and the identification of barriers preventing users from wearing their device. The step count and activity data will be reviewed to determine if users are continuing to wear their devices on a regular basis. In addition, the pre- and post- data collection surveys will collect user's impressions regarding Fitbit use and will address questions such as why users stop wearing their devices, how long users wear their devices, and how many devices are lost, destroyed, or not worn.

### Analysis

Cessation of tracker use over 4 months will be analyzed using survival analyses. Kaplan-Meier life table estimates will be used to characterize and visualize drop-offs in tracker use. This will show how the probability of drop-off in tracker use changes over time. Analyses will be done using the survival package and visualized using the survminer package in R [16-18]. The Kaplan-Meier plot will show cumulative survival probability, and the life table will show the number of participants at risk for dropping off of tracker use at multiple equally-spaced time intervals. The individual student surveys will also be reviewed to evaluate barriers hindering the student’s continued Fitbit usage.

### Ethics and Confidentiality

The IRB, through RTI International, was utilized to review and provide approval for the ActiviTeen study. Any further changes or modifications to the ActiviTeen study will be submitted as an amendment for IRB review. If any data breeches or adverse events occur throughout the study, the research team will ensure adequate documentation and notification of the IRB.

Confidentiality for the ActiviTeen study is through an RTI International guarantee only. Participating students were made aware within the assent document that the Fitbit app is not secure or encrypted. Anyone who has access to the student participant’s personal mobile phone may also have access to their collected activity data. Neither RTI International nor any member of ActiviTeen research team will retain the emails of student participants or their parents/guardians. No personally identifiable information will be connected to any collected data.

## Results

We are anticipating results for ActiviTeen by the end of 2016.

## Discussion

### Study Strengths

ActiviTeen provides a novel methodology for performing data collection utilizing a consumer wearable device. The Fitbit Zip is commercially available, inexpensive, and easily acquired by researchers. In addition, the study provides an overview for the steps required to collect participant’s PA data within an academic setting. This protocol is not meant to compare the Fitbit Zip to other devices (ie, accelerometer). The ActiviTeen protocol provides researchers with the necessary background to employ non-traditional devices for the collection of participant’s PA data (ie, Fitbit Zip).

### Limitations

ActiviTeen may have limited generalizability outside of Fitbit devices since the only consumer wearable device being utilized is the Fitbit Zip. However, the study should have generalizability to the wide range of products offered by Fitbit, since all appear to use the same online account interface. Furthermore, Research Triangle High School may be more accepting of technology-based studies when compared to other schools given it has been identified as a Science, Technology, Engineering, and Math focused institution. Since Research Triangle High School has also been classified as a charter school, the ActiviTeen staff did not have to receive approval from a pre-designated school board.

While creating, managing, and downloading data from 30 Fitbit accounts for the ActiviTeen pilot study is feasible, expanding the study to include a large population might prove problematic. The Fitbit account initiation process as well as downloading activity data and step count from more than 30 participants might likely prove time consuming and error prone. Expansion of the study in future work will require an alternative means of device set-up and data extraction.

### Conclusion

This research protocol has provided a detailed description of the steps that will be utilized to deploy the Fitbit Zip within an educational setting. This innovative method will be used to evaluate the feasibility of data collection with consumer wearable devices, the rate of attrition among users of consumer wearable devices, and the barriers preventing users from wearing their devices. We hope this protocol serves as a foundation for the implementation of other consumer wearable device driven studies.

## Abbreviations

IRB: institutional review board
METs: metabolic equivalents
PA: physical activity
